# Is Perioperative Dexmedetomidine Associated With a Reduced Risk of Perioperative Neurocognitive Disorders Following Cardiac Surgery? A Systematic Review and Meta-Analysis With Trial Sequential Analysis of Randomized Controlled Trials

**DOI:** 10.3389/fmed.2021.645975

**Published:** 2021-09-29

**Authors:** Xinglong Xiong, Dongxu Chen, Jing Shi

**Affiliations:** ^1^Department of Anesthesiology, The Affiliated Hospital of Guizhou Medical University, Guiyang, China; ^2^Department of Anesthesiology, West China Hospital, Sichuan University, Chengdu, China

**Keywords:** dexmedetomidine, delirium, cognitive dysfunction, cardiac surgery, meta-analysis

## Abstract

**Background:** To assess the effect of dexmedetomidine on the reducing risk of perioperative neurocognitive disorders (PNDs) following cardiac surgery.

**Methods:** A systematic review and meta-analysis with trial sequential analysis (TSA) of randomized controlled trials were performed. PubMed, Embase, Cochrane Library, and CNKI databases (to August 16, 2020) were searched for relevant articles to analyze the incidence of PND for intraoperative or postoperative dexmedetomidine administration after cardiac surgery. PND included postoperative cognitive dysfunction (POCD) and postoperative delirium (POD).

**Results:** A total of 24 studies with 3,610 patients were included. Compared with the control group, the incidence of POD in the dexmedetomidine group was significantly lower (odds ratio [OR]: 0.59, 95% CI: 0.43–0.82, *P* = 0.001), with firm evidence from TSA. Subgroup analyses confirmed that dexmedetomidine reduced the incidence of POD with firm evidence following coronary artery bypass grafting surgery (OR: 0.45, 95% CI: 0.26–0.79, *P* = 0.005), and intervention during the postoperative period (OR: 0.48, 95% CI: 0.34–0.67, *P* < 0.001). Furthermore, the incidence of POD in the dexmedetomidine group was also decreased in mixed cardiac surgery (OR: 0.68, 95% CI: 0.47–0.98, *P* = 0.039). Irrespective of whether “Confusion Assessment Method/Confusion Assessment Method for intensive care unit” or “other tools” were used as diagnostic tools, the results showed a decreased risk of POD in the dexmedetomidine group. There was no significant difference in the incidence of POCD (OR: 0.47, 95% CI: 0.22–1.03, *P* = 0.060) between the two groups, but this result lacked firm evidence from TSA.

**Conclusion:** The administration of dexmedetomidine during the perioperative period reduced the incidence of POD in patients after cardiac surgery, but there was no significant benefit in the incidence of POCD. The effect of dexmedetomidine on the incidence of POD or POCD following different types of surgery and the optimal dose and timing of dexmedetomidine warrant further investigation.

**Trial registration:** PROSPERO registration number: CRD42020203980. Registered on September 13, 2020.

## Introduction

Perioperative neurocognitive disorders (PNDs) include acute delirium and longer-lasting postoperative cognitive dysfunction (POCD) (1). Postoperative delirium (POD) and POCD have long been recognized as potential complications of anesthesia and surgery, with risk factors that include patient age, anesthetic drugs, and type of surgery (2, 3). The incidence of POD may vary depending on the type of surgery, with a previous study reporting an incidence of POD ranging from 3 to 47% following major cardiac surgery (4). Similarly, another study estimated the incidence of POD at 26–53% and 3-month POCD at about 10% (5). Further research confirmed that cardiac surgery was associated with higher rates of PND, prolonged length of hospitalization, and consequently increased burden of healthcare cost (6). Perhaps most concerning, POD and POCD have also been associated with long-term disability and increased mortality. Recognizing the significance of PND, the reduction of POD and POCD has been included as a target element of Enhanced Recovery After Surgery protocols (7). Though the pathogenesis of PND remains unclear, efforts to minimize the risk of POCD have taken on special importance.

Dexmedetomidine is a highly selective α_2_-adrenergic receptor agonist that has been widely used in the perioperative setting to provide sedation, anxiolysis, analgesia, and for its sympatholytic actions, which have been associated with neuroprotective effects and demonstrated to prevent the development of POD and POCD (8, 9). In contrast, a recently published study suggested that dexmedetomidine infusion did not decrease POD following cardiac surgery (10). Therefore, the neuroprotective effect of dexmedetomidine has been challenged and remains controversial, especially in cardiac surgery patients. The purpose of this meta-analysis of randomized controlled trials (RCTs) was to determine whether administration of dexmedetomidine reduced the incidence of PND following cardiac surgery.

## Methods

### This Meta-Analysis Was Conducted in Accordance With Cochrane Review

This systematic review and meta-analysis were conducted according to the Preferred Reporting Items for Systematic Reviews and Meta-Analyses statement (11). All analyses were made based on previously published studies; therefore, no ethical approval or patient consent was required. This study was registered in the international prospective register of systematic reviews (CRD42020203980).

### Eligibility Criteria

Included studies were limited to RCTs in adult surgical patients (age ≥ 18 years) that addressed the incidence of POD/POCD, administered dexmedetomidine, and were published from the inception of databases through August 16, 2020. Non-cardiac surgery, non-intravenous administration of dexmedetomidine, and animal experiments were excluded from this meta-analysis.

### Information Sources and Search

PubMed, Embase, Cochrane Library, and CNKI databases were systematically searched. Additional studies were identified from the reference sections of all eligible studies and previously published systematic reviews. According to the search strategy, both MeSH terms and free terms were used. A basic search strategy was conducted using the following terms: (dexmedetomidine OR “dexmedetomidine” [MeSH]) AND (perioperative neurocognitive disorders OR “perioperative neurocognitive disorders” [MeSH] OR PND) AND (postoperative cognitive dysfunction OR “postoperative cognitive dysfunction” [MeSH] OR POCD) AND (postoperative delirium OR “postoperative delirium” [MeSH] OR POD). A summary of the search strategies is shown in Supplementary 1.

### Data Extraction and Quality Assessment

**Figure 1:**
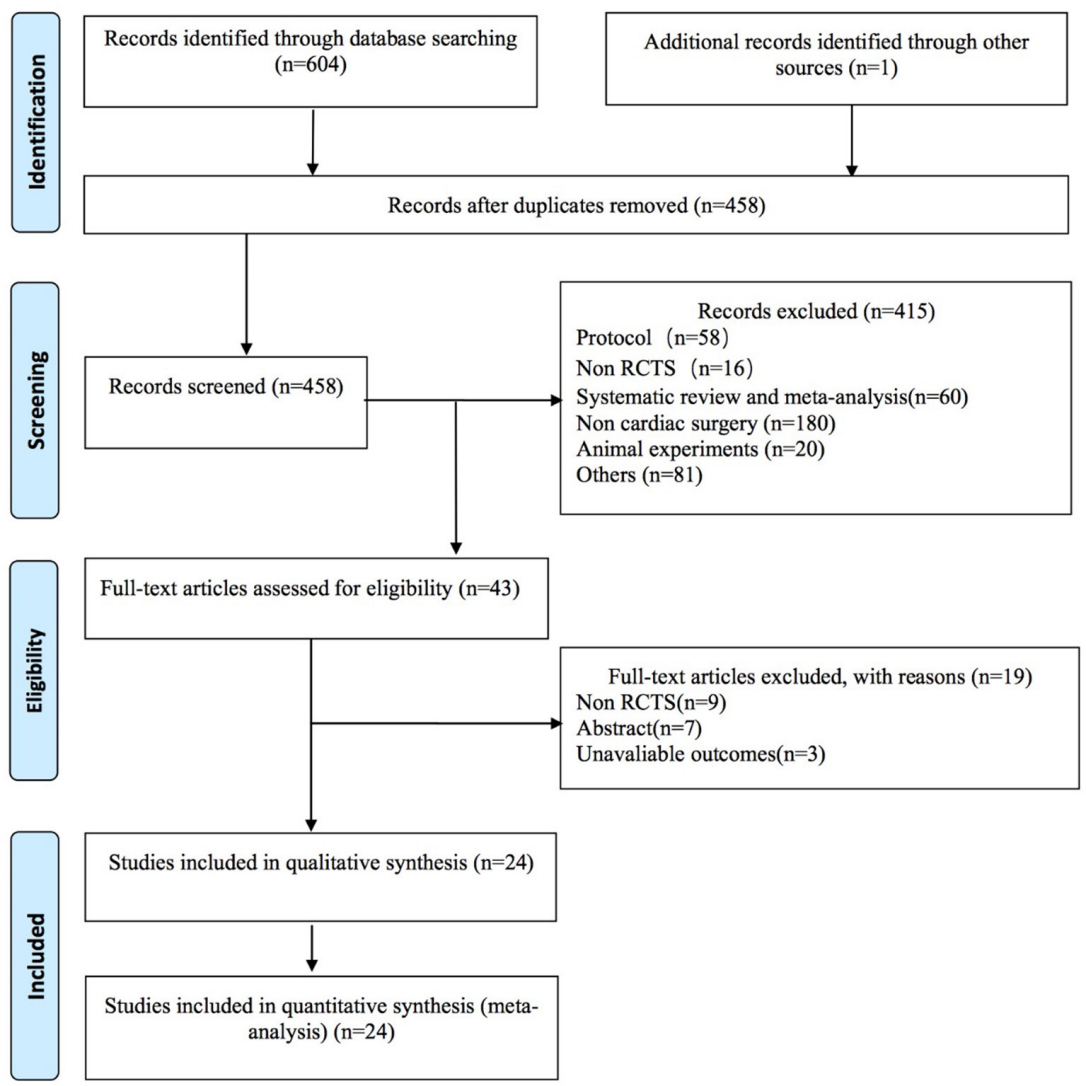
Preferred Reporting Items for Systematic Reviews and Meta-Analyses flow diagram for the literature search and exclusion criteria.

Data extraction and quality assessment were independently completed by two authors (XX and DC). Differences of opinion between the two authors were resolved by JS. Study elements included author, publication year, sample size, type of surgery, time and duration of the intervention or control group, the dosage of dexmedetomidine, POD/POCD assessment methods, and the incidence of POD/POCD. The risk of bias of the included studies was independently assessed by two reviewers (XLX and DXC). The Cochrane Collaboration Risk Assessment Tool (12) was adapted to evaluate the risk of bias for RCT evidence, seven domains of bias were classified as high, unclear, or low risk accordingly (Supplementary 2: Figure 1).

### Grading the Quality of Evidence

The quality of evidence for each finding was rated based on criteria established by the grading of recommendations assessment, development, and evaluation (GRADE) group (12). The quality of evidence was classified as very low, low, moderate, or high. Any disagreement was settled by discussion among the research team.

### Statistical Analysis

We performed the meta-analyses using Review Manager 5.3 (The Cochrane Collaboration, Copenhagen, Denmark) and STATA 15 software (Stata Corp LP, College Station, TX, USA). The odds ratios (OR) with 95% CIs were calculated for dichotomous outcomes. Considering the expected heterogeneity across studies, we applied a random-effects model to evaluate outcomes. We performed funnel plots to detect publication bias. *I*^2^ test was used to assess heterogeneity. Significant heterogeneity was denoted by *I*^2^ > 50%. To validate results, sensitivity analysis and subgroup analysis were performed. We conducted subgroup analysis based on the type of cardiac surgery, namely, cardiac valve surgery, mixed cardiac surgery, and coronary artery bypass grafting (CABG) surgery; time and duration of dexmedetomidine administration, categorized as the intraoperative period (dexmedetomidine infused from anesthesia induction to the end of surgery), perioperative period (dexmedetomidine infused from the surgical procedure and continued in the intensive care unit [ICU]), and postoperative period (dexmedetomidine infused in the ICU following cardiac surgery). Assessment for the diagnosis of delirium included the Confusion Assessment Method (CAM/CAM-ICU) or “other tools” that included the Richmond Agitation Sedation Scale (RASS), the modified Hewitt questionnaire, the Diagnostic and Statistical Manual of Mental Disorders (DSM-IV-TR), the Delirium Rating Scale (DRS), and the Intensive Care Delirium Screening Checklist (ICDSC). One trial diagnosed POD according to clinical criteria, and one study did not report their method of assessment. *P* < 0.05 was considered statistically significant.

Meta-analyses could be data driven because they were retrospectively conducted. Random errors could arise due to repetitive testings as data were accrued and testing of multiple outcome measures, which could lead to type I errors. To adjust for random error risk, meta-analyses (not reaching the required sample size) were analyzed with trial sequential monitoring boundaries (TSMBs) that are analogous to interim monitoring boundaries in a single trial. TSMBs adjusted the *P*-value that was required to reach statistical significance according to the number of participants and events in a meta-analysis. The fewer participants and events, the more restrictive the monitoring boundaries were, and the lower *P*-value was required to obtain statistical significance (13). Therefore, trial sequential analysis (TSA) offered the possibility to evaluate the credibility of the statistical results from our meta-analyses to decide whether CI and *P*-values in the meta-analyses were sufficient to show the anticipated effect. We calculated the required information size (IS) adjusted for the present meta-analysis and TSMBs to determine whether the evidence in our meta-analysis was reliable (14). If the cumulative Z-curve entered the futility area, or crossed TSMB, or reached the IS, we determined that the result had reached the anticipated intervention effect and showed firm evidence. Otherwise, the evidence was rated as absent. We set effect measure “Odds Risk” and model as “Random-effect (Dersimonian-Laird)” in the TSA software version 0.9 beta software (Copenhagen Trial Unit, Centre for Clinical Intervention Research, Copenhagen, Denmark). A two-sided TSA was performed to maintain a risk of 5% for type I error and a power of 80%.

## Results

### Study Selection

The flowchart of selection processes is shown in Figure 1. Our initial search identified 605 studies. After removing duplicates, we screened 458 studies based on abstracts. In total, we preliminarily evaluated 43 full-text articles for eligibility. Ultimately, 24 studies were enrolled in our systematic review and meta-analysis.

### Study Characteristics/Participants

The recorded elements of the enrolled studies are presented in Table 1. In total, 3,610 patients were analyzed in this study, 1,807 patients received dexmedetomidine and 1,803 patients received saline or other drugs. The number of cases included in each study ranged from 55 to 794. Specific to our study design, four studies concentrated on cardiac valve surgery (24, 26, 28, 34), 15 studies included combined CABG and valve surgeries (10, 16–23, 25, 27, 29, 31, 33, 37), and the remaining five studies related to CABG (15, 30, 32, 35, 36). Dexmedetomidine was administered during the intraoperative period in six studies (26–29, 31, 35), the perioperative period in eight studies (10, 15, 23–25, 33, 34, 37), and the other 10 studies during the postoperative period (16–22, 30, 32, 36). CAM/CAM-ICU was used in nine studies as the diagnostic tool for POD (16, 18, 20, 23, 25, 30–33) and five studies used RASS (10, 17, 19, 21, 22). The following tools were used to diagnose POD in a single trial each: the modified Hewitt questionnaire (15), the DSM-IV-TR (24), the DRS (26), and the ICDSC (37). One trial diagnosed POD according to clinical criteria (29), and one study did not report their method of measurement (36). The incidence of POD was reported in 19 studies (10, 15–25, 29–33, 36, 37), four studies reported the incidence of POCD (27, 28, 34, 35), and one study reported the incidence of both (26). Dexmedetomidine was demonstrated to reduce the incidence of POCD in three of four studies (27, 34, 35), 14 studies reported that dexmedetomidine decreased the incidence of POD (16–21, 23, 24, 29, 30, 32, 33, 36, 37), and one study found that dexmedetomidine reduced the risk of both POD and POCD (26).

**Table 1:** Main characteristics of all included studies.

**References**	**Age/mean age (yr.)**	**Sample size**	**Type of surgery**	**Control**	**Time and duration of intervention or control**	**Dosage**	**POD/POCD assessment methods**	**Incidence of POD/POCD**
Turan et al. (10)	18–85 DEX:63 ± 11 NS:62 ± 12	794	Mixed cardiac surgery	NS	Start before the surgical incision until 24 h after the infusion began	DEX: started with 0.1 ug·kg^−1^·h^−1^, and increased to 0.2 ug·kg^−1^·h^−1^ at the end of bypass, then increased to 0.4 ug·kg^−1^·h^−1^ postoperatively	RASS	DEX:67/398NS:46/396
Corbett et al. (15)	≥18 DEX:63.6 ± 10.1 PRO:62.4 ± 10.7	89	CABG	Propofol	DEX: start after bypass and continued for up to 1 h postextubation PRO: discontinued before extubation	DEX: 1 μg·kg^−1^ loading dose intravenously administered over 15 min, followed by a 0.4μg·kg^−1^·h^−1^ intravenous infusionPRO: 5–75 μg·kg^−1^·min^−1^	Modified Hewitt questionnaire	DEX:1/43PRO:1/46
Shehabi et al. (16)	≥60 DEX:71.5 (66–76) MOR:71.0 (65–75)	299	Mixed cardiac surgery	Morphine	Start within 1 h of admission to the ICU until removal of chest drain, when ready to discharge from ICU	DEX: 0.1-0.7 μg·kg-^1^·h^−1^MOR: 10-70 μg·kg^−1^·ml^−1^	CAM-ICU	DEX:13/152MOR:22/147
Eremenko and Chemova (17)	>18 DEX:56.3 PRO:59.3	55	Mixed cardiac surgery	Propofol	Start after ICU arrival continued for a maximum period of 24h	DEX: 0.2-0.7 ug·kg^−1^·h^−1^PRO: 0.3-2 mg·kg-1·h-1	RASS	DEX:2/28PRO:7/27
Park et al. (18)	≥18 and ≤ 90 DEX:51.09 ± 16.10 REM:54.35 ± 13.97	142	Mixed cardiac surgery	Remifentanil	Start immediately after ICU arrival until discharged from ICU	DEX: 0.5 μg·kg^−1^ loading dose; maintenance dose: 0.2 to 0.8 μg·kg^−1^·h^−1^	CAM-ICU	DEX:6/67REM:17/75
Priye et al. (19)	>18 DEX:45.1 ± 14.7 NS:41.4 ± 11.9	64	Mixed cardiac surgery	NS	Start after arrival on ICU and last for 12 h	DEX: 0.4 ug·kg^−1^·h^−1^	RASS	DEX:1/32NS:5/32
Djaiani et al. (20)	≥60 DEX:72.7 ± 6.4 PRO:72.4 ± 6.2	183	Mixed cardiac surgery	Propofol	Upon admission to ICU until for extubation	DEX: 0.4 ug·kg^−1^ loading dose intravenously administered over 10–20 min, followed by a 0.2–0.7 μg·kg^−1^·h^−1^ intravenous infusionPRO: 25–50 μg·kg^−1^·min^−1^	CAM-ICU	DEX:16/91PRO:29/92
Liu et al. (21)	≥18 DEX:53 (48–63) PRO:55 (48–62)	61	Mixed cardiac surgery	Propofol	On arrival in the ICU until extubation	DEX: 0.2–1.5 μg·kg^−1^·h^−1^PRO: 5–50 μg·kg^−1^·min^−1^	RASS	DEX:0/29PRO:2/32
Azeem et al. (22)	≥60 DEX:65.3 ± 4.8 MOR+MID:66.7 ± 5.6	60	Mixed cardiac surgery	Morphine+ Midazolam	On arrival in the ICU until extubation	DEX: 0.4–0.7 ug·kg^−1^·h^−1^MOR: 10–50 ug·kg^−1^·h^−1^MID: 0.05–0.2 mg·kg^−1^ repeated as needed	RASS	DEX:1/30MOR+MID:2/30
Likhvantsev et al. (23)	>45 DEX:62.6 ± 6.7 NS:62.4 ± 7.2	169	Mixed cardiac surgery (16); cardiac valve surgery (58); CABG (95)	NS	Start at anesthesia induction and continued in the ICU until the beginning of ventilation weaning	DEX: in the surgery: 0.7 ug·kg^−1^·h^−1^, in the ICU: 0.4-1.4 ug·kg^−1^·h^−1^	CAM-ICU	DEX:6/84NS:16/85
Maldonado et al. (24)	18–90 DEX:55 ± 16 PRO:58 ± 18 MID:60 ± 16	90	Cardiac valve surgery	Propofol Midazolam	After successful weaning from CPB DEX: continued for a maximum period of 24 h PRO and MID: discontinued before extubation	DEX: 0.4 μg·kg^−1^ loading dose; maintenance dose: 0.2–0.7 μg·kg^−1^·h^−1^PRO: 20–50 μg·kg^−1^·min^−1^MID: 0.5–2 mg·h^−1^	DSM-IV-TR	DEX:1/30PRO:15/30MID:15/30
Li et al. (25)	≥60 DEX:66.4 ± 5.4 NS:67.5 ± 5.3	285	Mixed cardiac surgery	NS	Start once the intravenous access was established for 10 min until end of MV	0.6 μg·kg^−1^ loading dose; maintenance dose: 0.4 μg·kg^−1^·h^−1^ after surgery: 0.1 μg·kg^−1^·h^−1^	CAM and CAM-ICU	DEX:7/142NS:11/143
Shu et al. (26)	45–75 DEX:47.7 ± 8.7 NS:46.8 ± 7.4	60	Cardiac valve surgery	NS	Start form routine anesthesia induction until the end of surgery	DEX: 1.0 μg·kg^−1^ loading dose; maintenance dose: 0.5 μg·kg^−1^·h^−1^	POCD: MMSE POD: DRS	POD:DEX:4/30NS:7/30POCD:DEX:4/30NS:12/30
Gong et al. (27)	DEX:42.3 ± 1.6 NS:42.4 ± 1.5	80	Mixed cardiac surgery	NS	Start after induction of anesthesia until the end of surgery	1 μg·kg^−1^ during the first 10 min, followed by the dose of 0.2 μg·kg^−1^	MMSE and MoCA	DEX:1/40NS:10/40
Kang et al. (28)	45–65 DEX:54.9 ± 8.6 ISO:56.5 ± 6.9	97	Cardiac valve surgery	Isoflurane	Start from cardiopulmonary bypass until the end of surgery	0.6 μg·kg^−1^ loading dose for 15 min; maintenance dose: 0.2 μg·kg^−1^·h^−1^	Antisaccadic eye movement test	DEX:11/50ISO:7/47
Sheikh et al. (29)	≤ 60 DEX:33.6 ± 11.82 PRO:35.56 ± 9.54	60	Mixed cardiac surgery	Propofol	Start after induction of anesthesia until skin closure	DEX: 1 μg·kg^−1^ loading dose; maintenance dose: 0.2–0.6 μg·kg^−1^·h^−1^PRO: 0.25–1 mg·kg^−1^·h^−1^	According to the pre-defined definition	DEX:1/30PRO:7/30
Massoumi et al. (30)	40–80 DEX:61.80 ± 7.90 NS:61.3 ± 8.90	88	CABG	NS	Start upon admission to ICU until for extubation	DEX: 1 μg·kg^−1^ loading dose; maintenance dose: 0.2–0.7μg·kg^−1^·h^−1^	CAM-ICU	DEX:4/44NS:9/44
Shi et al. (31)	≥60 DEX:74.7 ± 7.2 PRO:74.2 ± 7.7	164	Mixed cardiac surgery	Propofol	Start after anesthesia induction until the end of surgery	DEX: 0.4–0.6 μg·kg^−1^·h^−1^PRO: 50–80 mg·kg^−1^·h^−1^	CAM-ICU	DEX:33/84PRO:21/80
Shokri and Ali (32)	60–70 DEX:63.75 ± 3.29 CLO:64.38 ± 4.81	286	CABG	Clonidine	Start after ICU arrival DEX: continued for a maximum period of 72 h CLO: continued throughout MV	DEX: 0.7–1.4 μg·kg^−1^·h^−1^CLO: 0.5 μg·kg^−1^ loading dose; maintenance dose: 1–2 μg·kg^−1^·h^−1^	CAM-ICU	DEX:12/144CLO:23/142
Subramaniam et al. (33)	≥60 DEX:66.5 PRO:70.5	120	Mixed cardiac surgery	Propofol	Start during chest closure was continued for up to 6 h postoperatively or until extubation	DEX: 0.5–1 μg·kg^−1^ loading dose; maintenance dose: 0.1 to 1.4 μg·kg^−1^·h^−1^PRO: 20–100 ug·kg^−1^·min^−1^	CAM and CAM-ICU	DEX:10/59PRO:13/61
Zhou et al. (34)	>60 and ≤ 80 DEX:69.8 ± 5.1 NS:70.0 ± 4.9	76	Cardiac valve surgery	NS	DEX: start from induction to 2 h before extubation	DEX: 0.4 μg·kg^−1^·h^−1^	MoCA	DEX:6/38NS:12/38
Gao et al. (35)	65–70 DEX:69.5 ± 5.1 NS:70.4 ± 4.2	60	CABG	NS	Start at 15 min before incision until the end of the operation	DEX: 1 μg·kg^−1^ loading dose; maintenance dose: 0.3–0.5 μg·kg^−1^·h^−1^	MMSE	DEX:10/30NS:30/30
Balkanay et al. (36)	>18 60.5 ± 8.6	88	CABG	NS	Start after ICU arrival and continued for a maximum period of 24 h	0.04–0.50 μg·kg^−1^·h^−1^	Not reported	DEX:0/60NS:1/28
Li et al. (37)	51–78 DEX:64.1 ± 13.1 PRO:62.4 ± 11.9	140	Mixed cardiac surgery	PRO	Start from anesthesia induction to discharged from ICU	DEX: 1 μg·kg^−1^ loading dose; maintenance dose: 0.2–0.8 μg·kg^−1^·h^−1^PRO: 0.5 mg·kg^−1^ loading dose; maintenance dose: 0.5–1 mg·kg^−1^·h^−1^	ICDSC	DEX:8/72PRO:11/68

*Data presented as mean ± SD or median (interquartile range); CABG, coronary artery bypass grafting; CAM, Confusion Assessment Method; CAM-ICU, CAM for intensive care unit; CPB, cardiopulmonary bypass; CLO, clonidine; DEX, dexmedetomidine; DRS, Delirium Rating Scale; DSM-IV-TR, Diagnostic and Statistical Manual of Mental Disorders; ICDSC, Intensive Care Delirium Screening Checklist; ISO, isoflurane; MID, midazolam; MMSE, Mini Mental State Examination; MoCA, Montreal Cognitive Assessment; MOR, morphine; MV, mechanical ventilation; NS, normal saline; POCD, postoperative cognitive dysfunction; POD, postoperative delirium; PRO, propofol; RASS, Richmond Agitation Sedation Scale; REM, remifentanil*.

### Risk of Bias and Quality of Evidence

The overall quality of the studies was high. Seven domains of bias were described in Supplementary 2: Figure 1. There were no important imbalances at baseline in enrolled trials. None of the RCTs reported a loss of follow-up > 15%. GRADE evidence for POD and POCD is summarized in Supplementary 3.

### Outcomes

#### The Incidence of POD

**Figure 2:**
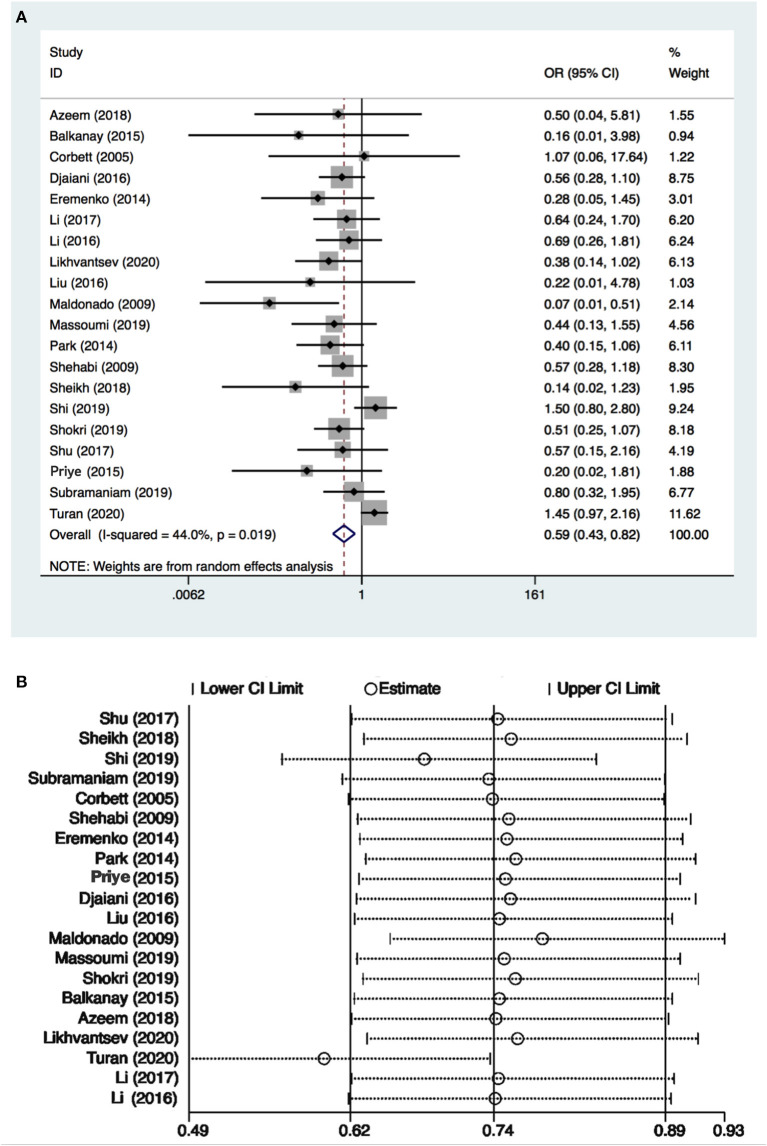
Postoperative delirium (POD) incidence and sensitive analysis within cardiac surgery. **(A)** Forest plot with POD incidence and **(B)** sensitive analysis for POD incidence. OR, odds ratio.

**Figure 3:**
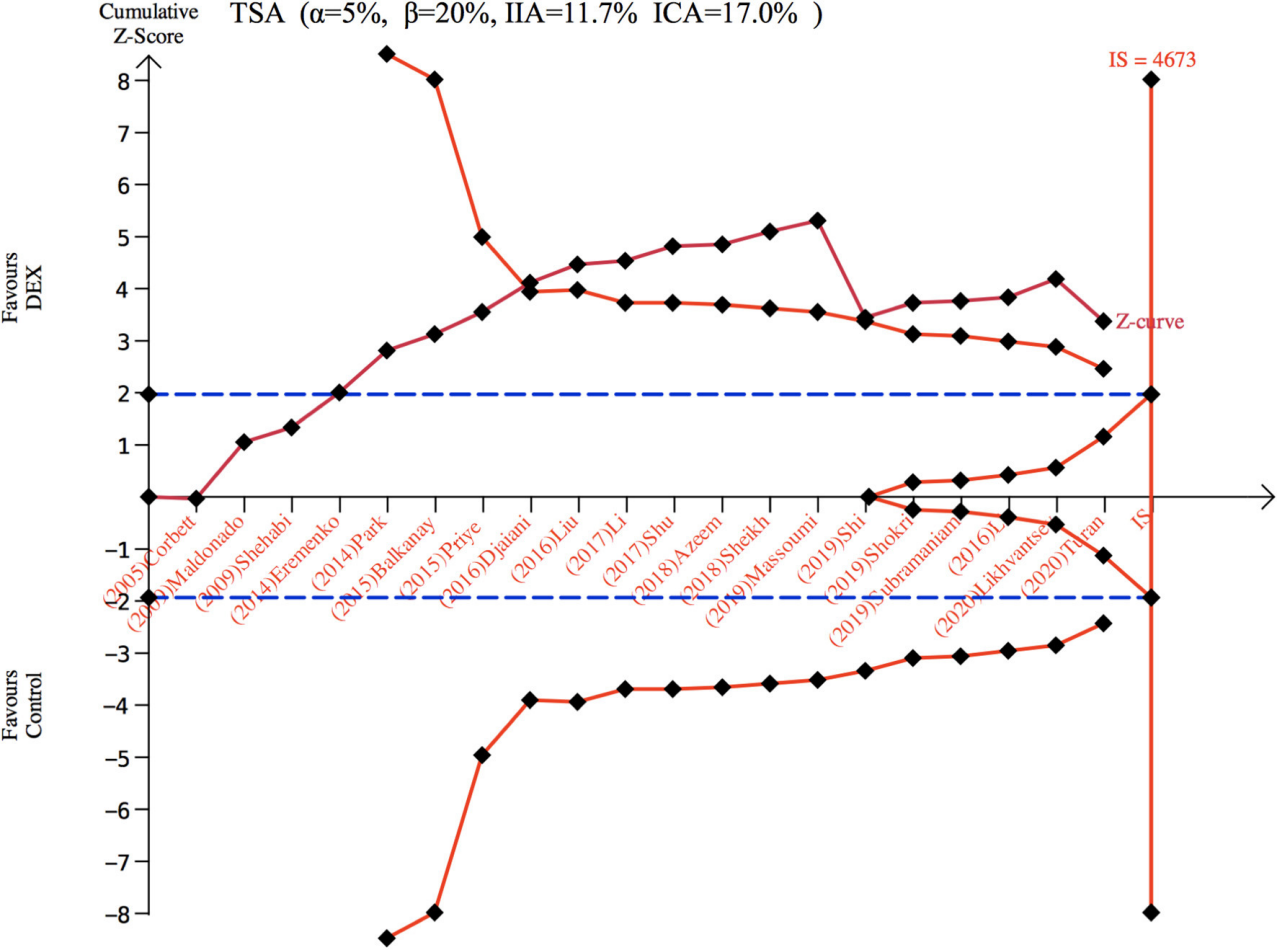
Trial sequence analysis for postoperative delirium. DEX, dexmedetomidine; ICA, incidence in control arm; IIA, incidence in intervention arm; IS, information size; TSA, trial sequential analyses.

There were 3,297 patients from 20 studies (10, 15–26, 29–33, 36, 37) included in the meta-analysis for POD. The incidence of POD in the dexmedetomidine group was significantly lower than in the control group (OR: 0.59, 95% CI: 0.43–0.82, *P* = 0.001; Figure 2A), without substantial heterogeneity (*I*^2^ = 44%). The funnel plot for the incidence of POD did not suggest publication bias (Supplementary 2: Figure 2). Sensitivity analysis of the incidence of POD, by excluding each study individually, found that the outcome was consistent (Figure 2B). Although the TSA required the IS to be 4,673 patients and the cumulative Z-curve did not reach this number, the cumulative Z-curve did cross TSMB (Figure 3). Therefore, the TSA of the pooled meta-analysis demonstrated firm evidence for the anticipated intervention effect. GRADE evidence for POD incidence within all included studies was moderate, downgraded for “inconsistency” (Supplementary 3A).

**Figure 4:**
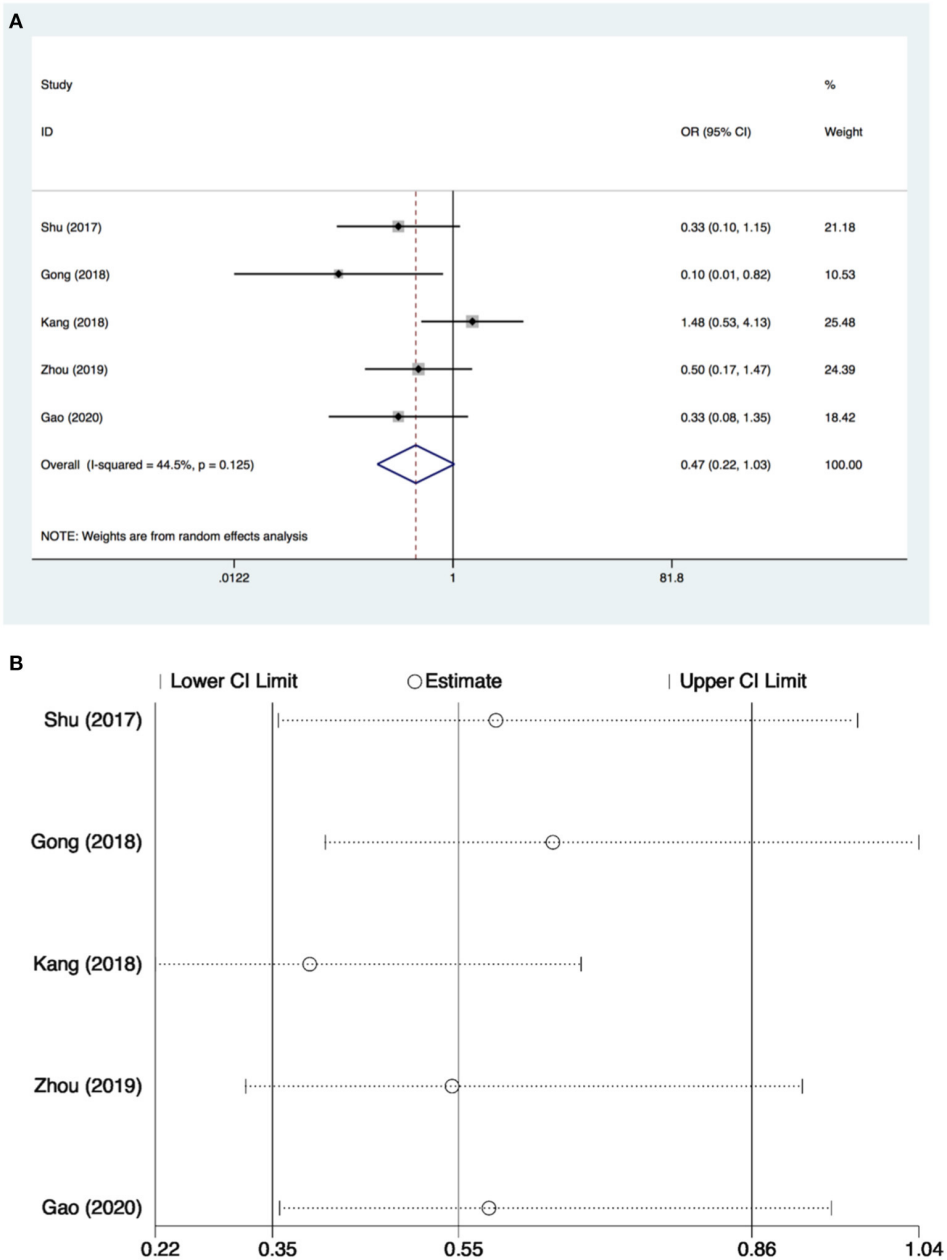
Postoperative cognitive dysfunction (POCD) incidence and sensitive analysis within cardiac surgery. **(A)** Forest plot with POCD incidence and **(B)** sensitive analysis for POCD incidence. OR, odds ratio.

Subgroup analyses were performed for different types of surgery, comprising three studies (23, 24, 26) with 208 patients conducted on cardiac valve surgery, 14 studies (10, 16–23, 25, 29, 31, 33, 37) with 2,443 patients focused on mixed cardiac surgery, and five studies (15, 23, 30, 32, 36) with 646 patients regarding CABG surgery. The forest plot revealed that there was no significant difference in POD incidence in cardiac valve surgery (OR: 0.34, 95% CI: 0.08–1.45, *I*^2^ = 52.6%, *P* = 0.146), while the POD incidence of dexmedetomidine-treated patients was significantly lower in mixed cardiac surgery (OR: 0.68, 95% CI: 0.47–0.98, *I*^2^ = 44.4%, *P* = 0.039) and CABG surgery (OR: 0.45, 95% CI: 0.26–0.79, *I*^2^ = 0.0%, *P* = 0.005) (Supplementary 2: Figure 4A). TSA analysis showed that the number of participants did not reach the IS in the “cardiac valve surgery” subgroup, but the Z-curve crossed the TSMB and futility boundary (FB) (Supplementary 2: Figure 4B). In the “mixed cardiac surgery” subgroup, TSA analysis revealed that the number of participants did not reach the IS or cross TSMB with the resultant absence of evidence for the anticipated intervention (Supplementary 2: Figure 4C). However, the dexmedetomidine-treated patients in the CABG group showed a decreased incidence of POD, and TSA revealed the required IS to be 405 patients. The cumulative Z-curve did reach the required IS, and TSA of the pooled meta-analysis confirmed firm evidence for the anticipated intervention effect (Supplementary 2: Figure 4D).

**Figure 5:**
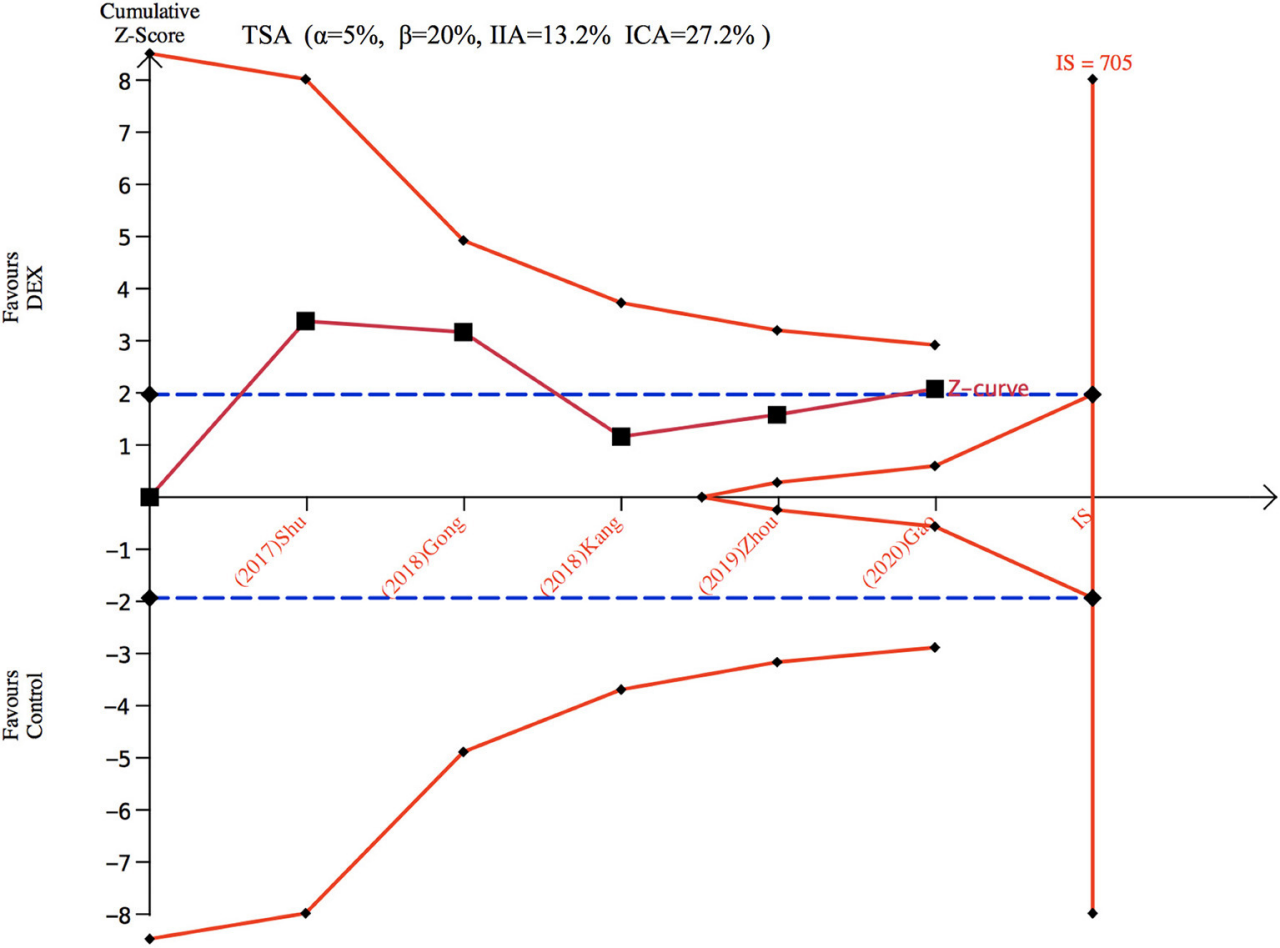
Trial sequence analysis for postoperative cognitive dysfunction (POCD). DEX, dexmedetomidine; ICA, incidence in control arm; IIA, incidence in intervention arm; IS, information size; TSA, trial sequential analyses.

Additional subgroup analyses were performed for different times (relative to the cardiac operations) and the duration of dexmedetomidine administration. These included three studies (26, 29, 31) involving 284 patients that were given dexmedetomidine during the intraoperative period; seven studies (10, 15, 23–25, 33, 37) with 1,687 patients treated in the perioperative period; while dexmedetomidine infusion postoperatively occurred in 10 studies (16–22, 30, 32, 36) with 1,326 patients. The forest plot revealed that there was no significant difference in POD incidence in the “intraoperative period” and the “perioperative period” subgroups; however, the POD incidence was significantly lower when dexmedetomidine was used during the postoperative period (OR: 0.48, 95% CI: 0.34–0.67, *I*^2^ = 0.0%, *P* < 0.001) (Supplementary 2: Figure 5A). TSA analysis showed that the number of participants did not reach the IS, but the Z-curve crossed FB when dexmedetomidine was used during the intraoperative period (Supplementary 2: Figure 5B). In the “perioperative period” subgroup, TSA analysis revealed that the number of participants did not reach the IS or cross TSMB with the resultant absence of evidence for the anticipated intervention (Supplementary 2: Figure 5C). In the “postoperative period” subgroup, TSA analysis showed that the number of participants reached the IS, and the Z-curve crossed TSMB and FB (Supplementary 2: Figure 5D).

Further subgroup analysis was conducted based on different diagnostic tools used to assess POD. These included nine studies (16, 18, 20, 23, 25, 30–33) with 1,736 patients that used CAM/CAM-ICU, whereas the remaining 11 studies (10, 15, 17, 19, 21, 22, 24, 26, 29, 36, 37) with 1,561 patients used other different measurements, labeled as “other tools.” The forest plot showed a statistical difference both in the subgroup using “CAM/CAM-ICU” (OR: 0.64, 95% CI: 0.47–0.87, *I*^2^ = 21.4%, *P* = 0.001) and “other tools” (OR: 0.44, 95% CI: 0.22–0.89, *I*^2^ = 52.7%, *P* = 0.023) (Supplementary 2: Figure 6A). TSA analysis showed that the number of participants did not reach the IS or cross TSMB in the “other tools” subgroup, indicating the absence of evidence for the anticipated intervention effect (Supplementary 2: Figure 6B). However, for the diagnostic tool CAM/CAM-ICU, although the cumulative Z-curve did not reach the required IS, the cumulative Z-curve crossed TSMB (Supplementary 2: Figure 6C) indicating that TSA of the pooled meta-analysis had firm evidence for the anticipated intervention effect.

#### The Incidence of POCD

Only five studies (26–28, 34, 35) with 373 patients evaluated the incidence of POCD. There was no significant difference found in the incidence of POCD for dexmedetomidine administration when compared with other drugs (OR: 0.47, 95% CI: 0.22–1.03, *I*^2^ = 44.5%, *P* = 0.060) (Figure 4A). The funnel plot for the total POCD incidence did not suggest the presence of publication bias (Supplementary 2: Figure 3). Sensitivity analysis of the incidence of POCD, by excluding each study individually, found the outcome was consistent (Figure 4B). TSA revealed that the required IS was 705 patients, but the cumulative Z-curve did not reach the required IS. TSA showed that the Z-curves did not cross the TSMB or the FB; therefore, there was an absence of evidence for the anticipated intervention effect (Figure 5). GRADE evidence for POCD incidence for all included studies was moderate, downgraded due to “small sample size” (Supplementary 3B).

Subgroup analysis was also performed according to different types of cardiac surgery (cardiac valve surgery, mixed cardiac surgery, and CABG surgery) and different intervention time points (intraoperative period and perioperative period). Only three studies (26, 28, 34) including 233 patients were identified in the cardiac valve surgery subgroup, one (27) with 80 patients for the mixed cardiac surgery subgroup, and one other study (35) including 60 patients for the CABG subgroup. Regarding operative time-point interventions, four studies (26–28, 35) with 297 patients contributed data to the intraoperative subgroup and one study (34) with 76 patients to the perioperative subgroup. The subgroup analyses showed a statistical difference in the “mixed cardiac surgery” group, but there was no significant difference in all of the other subgroups for incidence of POCD with dexmedetomidine intervention (Supplementary 2: Figures 7A, 8A). TSA analysis showed the number of participants did not reach the IS or cross TSMB in either subgroup, indicating the absence of evidence for anticipated intervention effect (Supplementary 2: Figures 7B, 8B). All outcomes of meta-analysis and trial sequential analysis are presented in Table 2.

**Table 2:** Meta-analysis and trial sequential analysis for subgroup analyses of postoperative delirium incidence and postoperative cognitive dysfunction.

**Outcomes**	**Meta-analysis**					**TSA**							
	**OR**	**95% CI**	***P*** *	***I*** **^2^**	***P*** **^#^**	**IIA%**	**ICA%**	**D^2^%**	**Required IS**	**Reach IS**	**Cross TSMB**	**Cross FB**	**Evidence**
POD incidence	0.59	0.43, 0.82	0.019	44	0.001	11.7	17.0	17.6	4,673	No	Yes	No	FE
POD incidence within cardiac valve surgery	0.34	0.08, 1.45	0.121	52.6	0.146	4.8	31.2	71.57	235	No	Yes	Yes	FE
POD incidence within mixed cardiac surgery	0.68	0.47, 0.98	0.037	44.4	0.039	13.6	16.1	71.72	22,465	No	No	No	AE
POD incidence within CABG	0.45	0.26, 0.79	0.864	0	0.005	5.9	14.3	0	405	Yes	No	Yes	FE
POD incidence when intervention given during intraoperative period	0.69	0.21, 2.29	0.068	62.9	0.538	26.4	25.0	85.14	22,465	No	No	Yes	FE
POD incidence when intervention given during perioperative period	0.66	0.36, 1.22	0.015	61.9	0.184	12.1	14.9	82.67	26,983	No	No	No	AE
POD incidence when intervention given during postoperative period	0.48	0.34, 0.67	0.984	0	<0.001	8.1	18.0	0	364	Yes	Yes	Yes	FE
POD incidence diagnosed with other tools	0.44	0.22, 0.89	0.020	52.7	0.023	11.0	15.3	82.25	12,316	No	No	No	AE
POD incidence diagnosed with CAM or CAM-ICU	0.64	0.47, 0.87	0.253	21.4	0.001	12.3	18.5	49.56	2,111	No	Yes	No	FE
POCD incidence	0.47	0.22, 1.03	0.125	44.5	0.060	13.2	27.2	63.27	705	No	No	No	AE
POCD incidence within cardiac valve surgery	0.66	0.27, 1.58	0.152	47	0.348	17.8	27	67.2	1,979	No	No	No	AE
POCD incidence within mixed cardiac surgery	0.10	0.01, 0.82	Cannot be calculated due to insufficient information										AE
POCD incidence within CABG	0.33	0.08, 1.35	Cannot be calculated due to insufficient information										AE
POCD incidence when intervention given during intraoperative period	0.44	0.15, 1.26	0.066	58.3		12.7	25.9	25.9	1,042	No	No	No	AE
POCD incidence when intervention given during perioperative period	0.50	0.17, 1.47	Cannot be calculated due to insufficient information										AE

* *P for heterogeneity*.

# *P for difference*.

*TSA, trial sequential analyses; OR, odds ratio; IIA, incidence in intervention arm; ICA, incidence in control arm; D^2^, diversity; IS, information size; TSMB, trial sequential monitoring boundary; FB, futility boundary; POD, postoperative delirium; CABG, coronary artery bypass surgery; FE, firm evidence; AE, absent evidence; CAM, confusion assessment method; CAM-ICU, CAM in the ICU; POCD, postoperative cognitive dysfunction*.

*Error a and 1-β were defined as 5 and 80%, respectively, in each model; IIA and ICA were calculated from the average incidence in the intervention group and the control group separately; D^2^ was autogenerated by the TSA software*.

## Discussion

This meta-analysis demonstrates that administration of dexmedetomidine could decrease the risk of POD for adult cardiac surgical patients, with firm evidence from TSA. However, dexmedetomidine did not reduce the incidence of POCD following cardiac surgery in a statistically significant way, but TSA suggested that this outcome lacked firm evidence.

Prior research indicated that cardiac surgery has been associated with higher rates of PND, a serious complication associated with high morbidity and mortality (38) and that the most promising pharmacological strategy to avoid this complication seemed to be perioperative administration of dexmedetomidine (9, 39). In recent years, a great deal of research confirmed that dexmedetomidine had a protective effect on multiple organ systems, namely, the heart, lungs, kidneys, liver, and the central nervous system. The reported neuroprotective mechanisms of dexmedetomidine included (1) inhibiting the excitability of sympathetic nerves and regulating the release of catecholamines; (2) regulating the release of central glutamate; (3) inhibiting cell apoptosis and release of inflammatory cytokines; (4) antioxidant stress; and (5) regulating synaptic plasticity and reducing neurotoxicity of anesthetics (40, 41). Our study showed a decreased risk of POD following dexmedetomidine administration in cardiac surgery. Although the TSA showed that Z-curves did not reach the required IS, since they did cross TSMB, statistical significance was reached to detect intervention effect for dexmedetomidine administration following cardiac surgery. Data from a recently published trial conducted by Turan et al. (10) conflicted with our results. A total of 798 patients who underwent cardiac surgery were included. In the placebo group, POD occurred with an incidence of 12% compared to an incidence of POD of 17% in patients who had received dexmedetomidine. The authors concluded that dexmedetomidine did not decrease POD in patients undergoing cardiac surgery, and dexmedetomidine should be used cautiously in cardiac surgical patients with attention to preventing hypotension. Another meta-analysis (42) assessed the effect of dexmedetomidine on POD in elderly cardiac surgical patients that included five studies with 1,217 patients, and also demonstrated that dexmedetomidine did not prevent POD, in recognition of the higher incidence of POD in elderly patients, the author suggested that the sample size may have contributed to the negative finding, a possibility that should be explored further. In contrast, a previous study had suggested that dexmedetomidine could reduce the risk of POD in non-cardiac surgery (43). In addition, Duan et al. (44) suggested that dexmedetomidine could reduce the incidence of POD in adult cardiac surgical patients, but no subgroup analysis was conducted to clarify whether the effect of dexmedetomidine on POD in cardiac surgical patients differed between different types of cardiac surgery or different time-points of dexmedetomidine administration. Considering the inconclusive and controversial results of prior studies, we performed an updated meta-analysis on this topic. According to the firm evidence of TSA and moderate quality of GRADE, we suggested that dexmedetomidine infusion was a reasonable pharmacological strategy for reducing the risk of POD in cardiac surgical patients. This conclusion was supported by the European Society of Anesthesiology and Intensive Care recommendation that dexmedetomidine might be considered to decrease the incidence of POD following cardiac or vascular surgery (45).

Considering the internal heterogeneity of the enrolled studies, we performed subgroup analyses to verify the consistency of the results. The result of our subgroup analysis for different types of cardiac surgery indicated that the incidence of POD could be decreased with dexmedetomidine administration in mixed cardiac surgery and CABG surgery, whereas there was no significant difference in cardiac valve surgery. The trials in the “mixed cardiac surgery” subgroup comprised studies without distinguishing specifically between CABG and cardiac valve surgeries, which would require the inclusion of additional numbers to detect a statistical difference. A previous study found that treatment with dexmedetomidine did significantly decrease the incidence of delirium following mixed cardiac surgery, whereas a similar difference was not apparent in the CABG group (46). These results were different from our study, but might be explained by the fact that only 2 of their 10 included studies focused on CABG surgery. The incidence of delirium following CABG was reported to be 30.52% (47), while patients after cardiac valve surgery were more likely to develop POD and POCD than after CABG surgery alone, perhaps contributing to other complications and reflective of a longer recovery period (48). Nevertheless, based on our results, dexmedetomidine did not prove statistically advantageous in reducing the incidence of POD for patients undergoing cardiac valve surgery. TSA revealed an absence of evidence for the anticipated intervention effect when dexmedetomidine was used in mixed cardiac surgery and cardiac valve surgery; additional studies are needed to further define the risks and benefits of dexmedetomidine in different types of cardiac surgery.

Based on different time-points of dexmedetomidine administration, subgroup analysis showed that the incidence of POD was significantly lower when dexmedetomidine was used during the postoperative period, but there were no significant differences in the “perioperative period” and the “intraoperative period” subgroups. Since only three studies were included in the “intraoperative period” subgroup, the results may have been influenced by the small sample size. A previous report (49) of dexmedetomidine infusion used as the primary or sole sedative in ICU patients did not lower 90-day mortality, coma, and delirium compared to usual care. On the contrary, our study demonstrated the prevention of POD during the postoperative period. Further research is needed to clarify the effect of perioperative dexmedetomidine administration on the incidence of POD.

Confusion Assessment Method for intensive care unit is known for its high validity and reliability for the detection of ICU delirium (81% sensitivity and 96% specificity) (50). In this study, nine studies applied the CAM or CAM-ICU to detect POD, and 11 studies used “other tools.” Our results showed a decrease in the incidence of POD whether CAM/CAM-ICU or “other tools” were used as the diagnostic tool. Based on the firm evidence for the anticipated intervention effect from TSA, CAM, and CAM-ICU were further verified as valid tools for the diagnosis of delirium.

Regarding our investigation of the incidence of POCD after dexmedetomidine administration in cardiac surgery, there was no significant difference between dexmedetomidine administration compared with other drugs. Contrary to our results, four of the five studies (26, 27, 34, 35) included in our meta-analysis suggested that dexmedetomidine decreased the incidence of POCD. Only one study (28) reported a different outcome. This study enrolled 97 patients, and dexmedetomidine infusion during cardiac valve surgery with cardiopulmonary bypass decreased the concentrations of biochemical markers of brain injury (matrix metalloproteinase-9 and glial fibrillary acidic protein) but did not improve POCD in the early postoperative period. All five studies had followed up for 7 days after the surgery to measure POCD, but other studies have shown that the incidence of POCD in cardiac surgery patients 1 month postoperatively ranged from 12 to 30% (51). In our study, dexmedetomidine infusion was limited to the intraoperative period in four studies, and the perioperative period in one study. For a beneficial effect of dexmedetomidine on POCD after cardiac surgery, a continuous infusion might be necessary. More high-quality studies with larger sample sizes are needed.

In further subgroup analyses based on different types of cardiac surgery and different intervention time points of dexmedetomidine infusion, except for a decrease in POCD with dexmedetomidine administration in the mixed cardiac surgery group, no other subgroup analysis showed a statistically significant difference. Because few available studies were included in these subgroups, the number of trials and patients was markedly low in the subgroup analysis. The maintenance dose of all the studies (26–28, 35) in the intraoperative period subgroup ranged between 0.2 and 0.5 ug·kg^−1^·h^−1^, which was lower than the recommended maximum sedation dosage and may have affected the results. Therefore, it is impossible to draw meaningful conclusions from these results. Many publications have indicated favorable outcomes of dexmedetomidine for the reduction of POD, but further studies are needed before recommending the use of dexmedetomidine for reduction of POCD (52), particularly for patients undergoing cardiac surgery.

### Limitations

Several limitations to the present meta-analysis need to be considered in the interpretation of our results. First, the sample size of this meta-analysis is relatively small, therefore, at potential risk of inaccurately estimating treatment effects. Second, the duration and dosage of dexmedetomidine varied markedly between studies which may have influenced the results. Third, some of the analyses were limited by underpowered statistics, namely, heterogeneity in the characteristics of the participants (e.g., underlying diseases, the type of surgery, initial severity of PND, and trial duration), the small trial numbers for some treatment arms, heterogeneous diagnostic assessment tools, and the inclusion of few studies on the influence of different interventions for the treatment and prevention of PND.

## Conclusion

In summary, the administration of dexmedetomidine during the perioperative period reduced the incidence of POD in patients following cardiac surgery, but there was no significant reduction in the incidence of POCD. Further research is needed to explore the neuroprotective effect of perioperative dexmedetomidine, particularly regarding the optimal dose, timing of administration, and need for maintenance infusion.

## Data Availability Statement

The original contributions presented in the study are included in the article/Supplementary Material, further inquiries can be directed to the corresponding author/s.

## Author Contributions

XX, DC, and JS conceived and designed the work, developed the study design, were involved with data acquisition and interpretation, drafted the manuscript, edited the draft, and approved the final version. All authors contributed to the article and approved the submitted version.

## Funding

This work was supported by the Science and Technology Project of Guizhou Provincial Health Commission (No. gzwjkj2019-1-160) and the Research Fund for the Doctoral Program of The Affiliated Hospital of Guizhou Medical University (No. I-2019-03).

## Conflict of Interest

The authors declare that the research was conducted in the absence of any commercial or financial relationships that could be construed as a potential conflict of interest.

## Publisher's Note

All claims expressed in this article are solely those of the authors and do not necessarily represent those of their affiliated organizations, or those of the publisher, the editors and the reviewers. Any product that may be evaluated in this article, or claim that may be made by its manufacturer, is not guaranteed or endorsed by the publisher.
