# Supplementary material for: Is Perioperative Dexmedetomidine Associated With a Reduced Risk of Perioperative Neurocognitive Disorders Following Cardiac Surgery? A Systematic Review and Meta-Analysis With Trial Sequential Analysis of Randomized Controlled Trials
Source: Front Med (Lausanne). 2021 Sep 29;8:645975. doi: 10.3389/fmed.2021.645975 (PMC8511308; doi:10.3389/fmed.2021.645975)
Supplement: Supplementary file 1 [file Data_Sheet_1.docx]

**Supplementary 1. Search Strategy**

PubMed Search Strategy inception up to August 13, 2020

#1 Search "dexmedetomidine"[Title/Abstract]

#2 perioperative neurocognitive disorders [MeSH Terms]

#3 ((#2) OR (perioperative neurocognitive disorders [Title/Abstract])) OR (PND[Title/Abstract])

#4 postoperative cognitive dysfunction [MeSH Terms]

#5 ((#4) OR (POCD[Title/Abstract])) OR (postoperative cognitive dysfunction [Title/Abstract])

#6 postoperative delirium [MeSH Terms]

#7 ((#6) OR (postoperative delirium [Title/Abstract])) OR (POD[Title/Abstract])

#8 ((#3) OR (#5) OR (#7))

#9 ((#1) AND (#8))

Cochrane Library Search Strategy inception up to August 15, 2020

#1 MeSH descriptor: [Dexmedetomidine] this term only

#2 (dexmedetomidine):ti,ab,kw

#3 #1 or #2

#4 (perioperative neurocognitive disorders):ti,ab,kw OR (PND):ti,ab,kw

#5 MeSH descriptor: [Postoperative Cognitive Complications] this term only

#6 (Postoperative Cognitive Complications):ti,ab,kw

#7 (postoperative cognitive dysfunction):ti,ab,kw OR (POCD):ti,ab,kw

#8 #5 or #6 or #7

#9 (postoperative delirium):ti,ab,kw OR (POD):ti,ab,kw

#10 #4 or #8 or #9

#11 #3 and #10

Ovid (EMBASE and MEDLINE) inception up to August 16, 2020

Ovid (EMBASE and MEDLINE)

#1 dexmedetomidine.m_titl.

#2 limit 1 to abstracts

#3 perioperative neurocognitive disorders.m_titl.

#4 limit 3 to abstracts

#5 PND.m_titl.

#6 limit 5 to abstracts

#7 postoperative cognitive dysfunction.m_titl.

#8 limit 7 to abstracts

#9 POCD.m_titl.

#10 limit 9 to abstracts

#11 postoperative delirium.m_titl.

#12 limit 11 to abstracts

#13 POD.m_titl.

#14 limit 13 to abstracts

#15 4 or 6

#16 8 or 10

#17 12 or 14

#18 15 or 16 or 17

#19 2 and 18
