# Supplementary material for: Is Perioperative Dexmedetomidine Associated With a Reduced Risk of Perioperative Neurocognitive Disorders Following Cardiac Surgery? A Systematic Review and Meta-Analysis With Trial Sequential Analysis of Randomized Controlled Trials
Source: Front Med (Lausanne). 2021 Sep 29;8:645975. doi: 10.3389/fmed.2021.645975 (PMC8511308; doi:10.3389/fmed.2021.645975)
Supplement: Supplementary file 2 [file Data_Sheet_2.docx]

***Supplementary 2: Supplementary Figures***

Fig S1. (A) Risk of bias summary: review authors’ judgements about each risk of bias item for each included study. (B) Risk of bias graph: review authors’ judgements about each risk of bias item presented as percentages across all included studies.


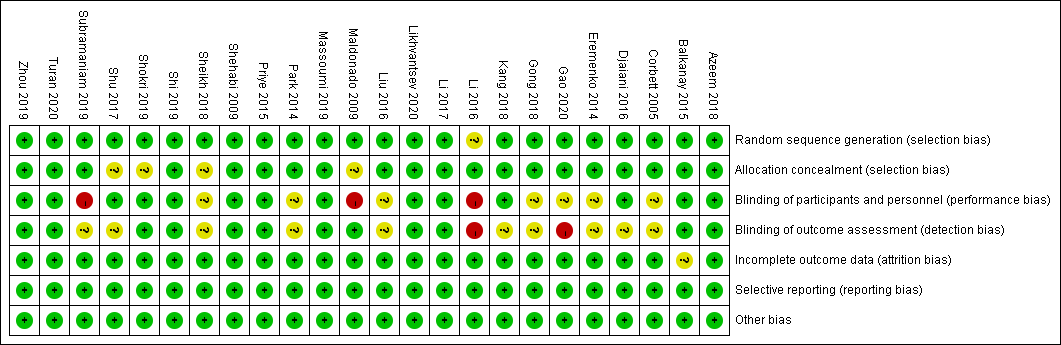
A.

B.


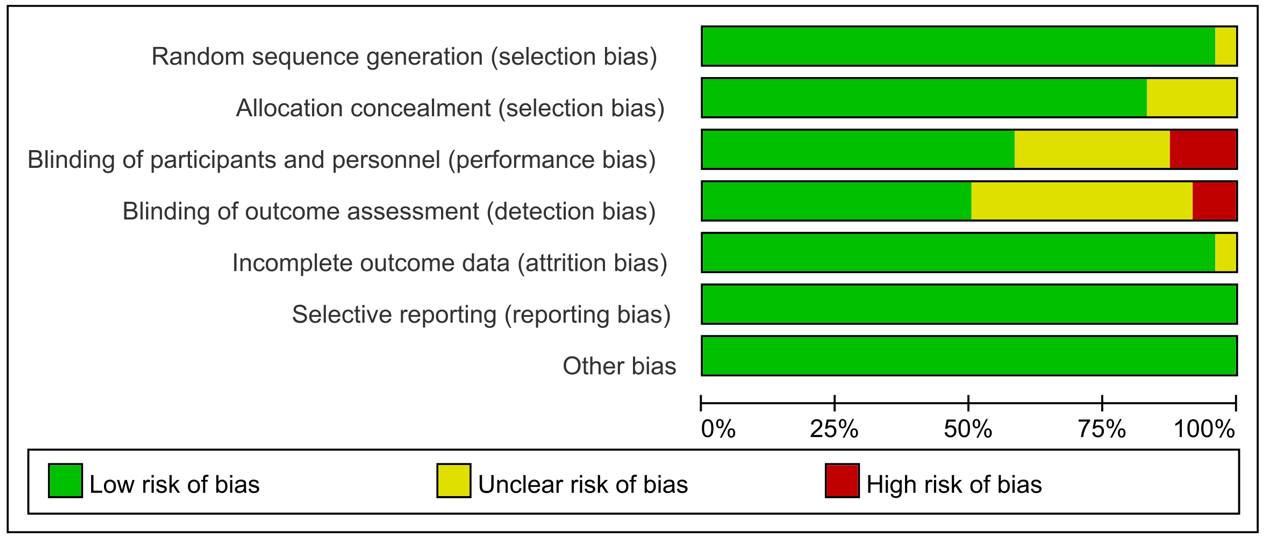


Fig S2. Funnel plot for the total postoperative delirium incidence did not suggest the presence of publication bias.

NOTE: OR, odds ratio.


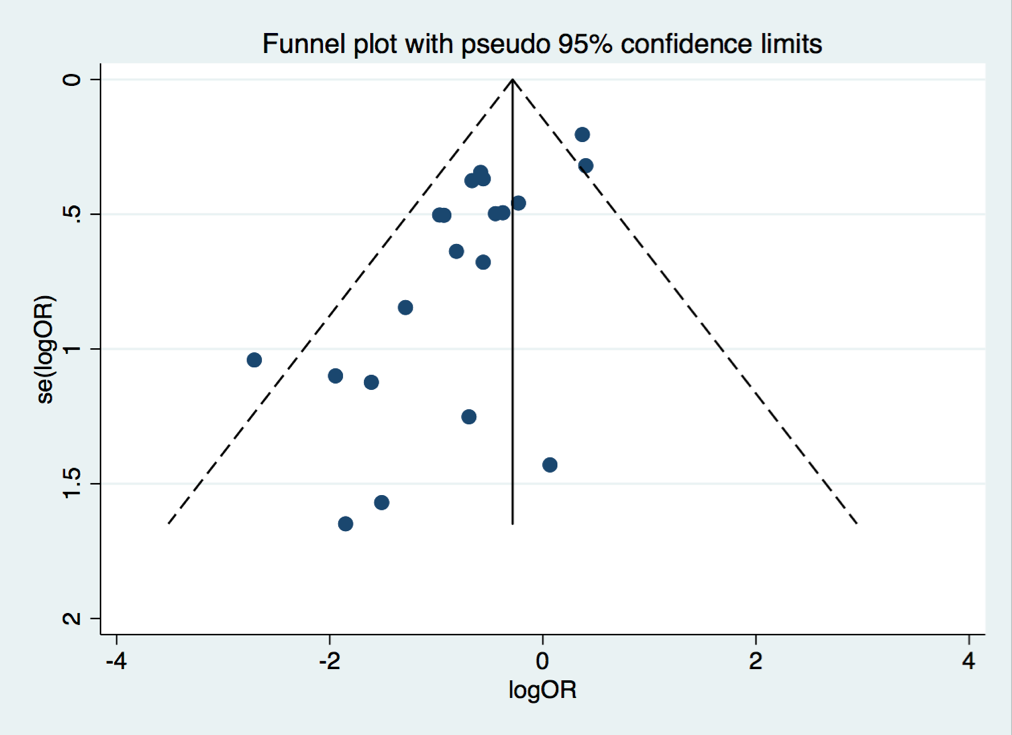


Fig S3. Funnel plot for the total postoperative cognitive dysfunction did not suggest the presence of publication bias.

NOTE: OR, odds ratio.

**
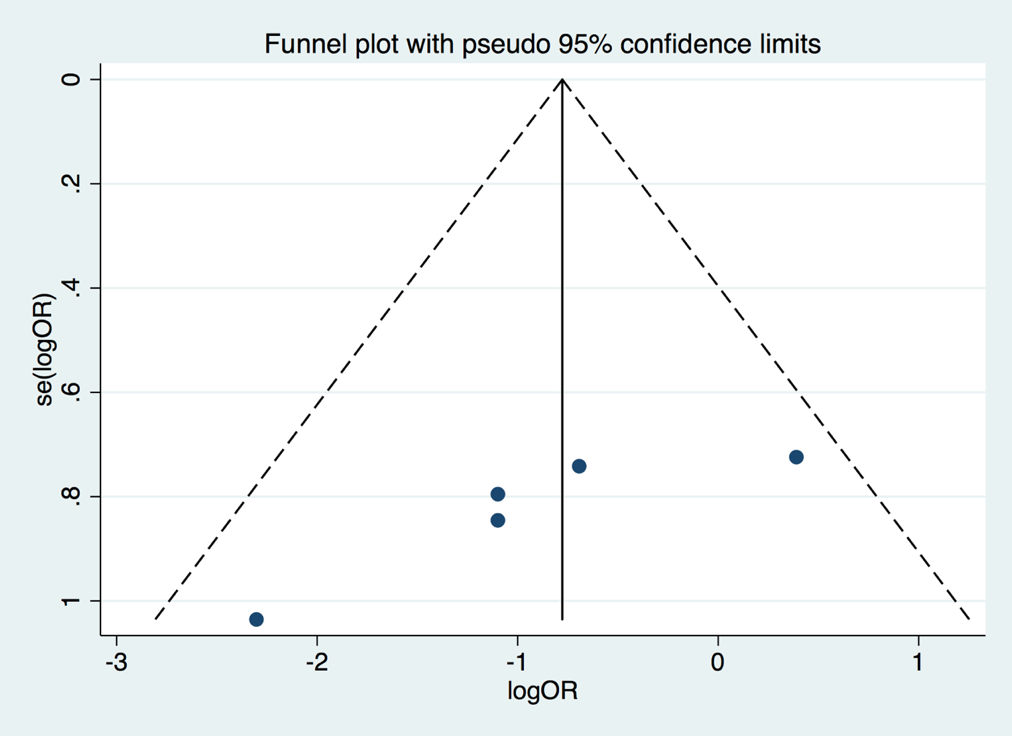
**


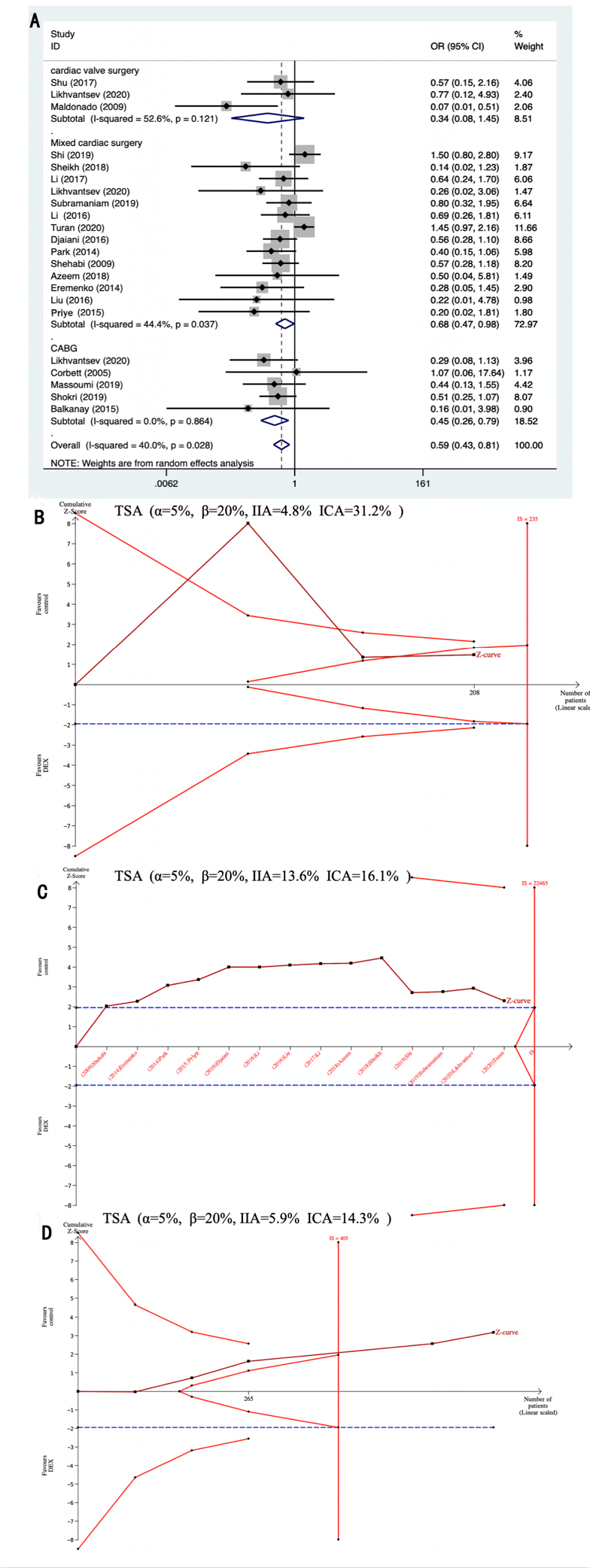
Fig S4. Postoperative delirium incidence depending on the type of surgery and trial sequential analyses. (A) Forest plot with subgroup analysis depending on the type of surgery. (B) Trial sequential analyses for ‘cardiac valve surgery’ subgroups: error α=5%, β=20%, IIA=4.8%, ICA=31.2%. (C) Trial sequential analyses for ‘Mixed cardiac surgery’ subgroups: error α=5%, β=20%, incidence in intervention arm (IIA)=13.6%, incidence in control arm (ICA)=16.1%. (D) Trial sequential analyses for ‘CABG surgery’ subgroups: error α=5%, β=20%, IIA=5.9%, ICA=14.3%.

NOTE: OR, odds ratio; CI, conﬁdence intervals; CABG, coronary artery bypass surgery; TSA, trial sequential analyses; IIA, incidence in intervention arm; ICA, incidence in control arm; IS, information size.


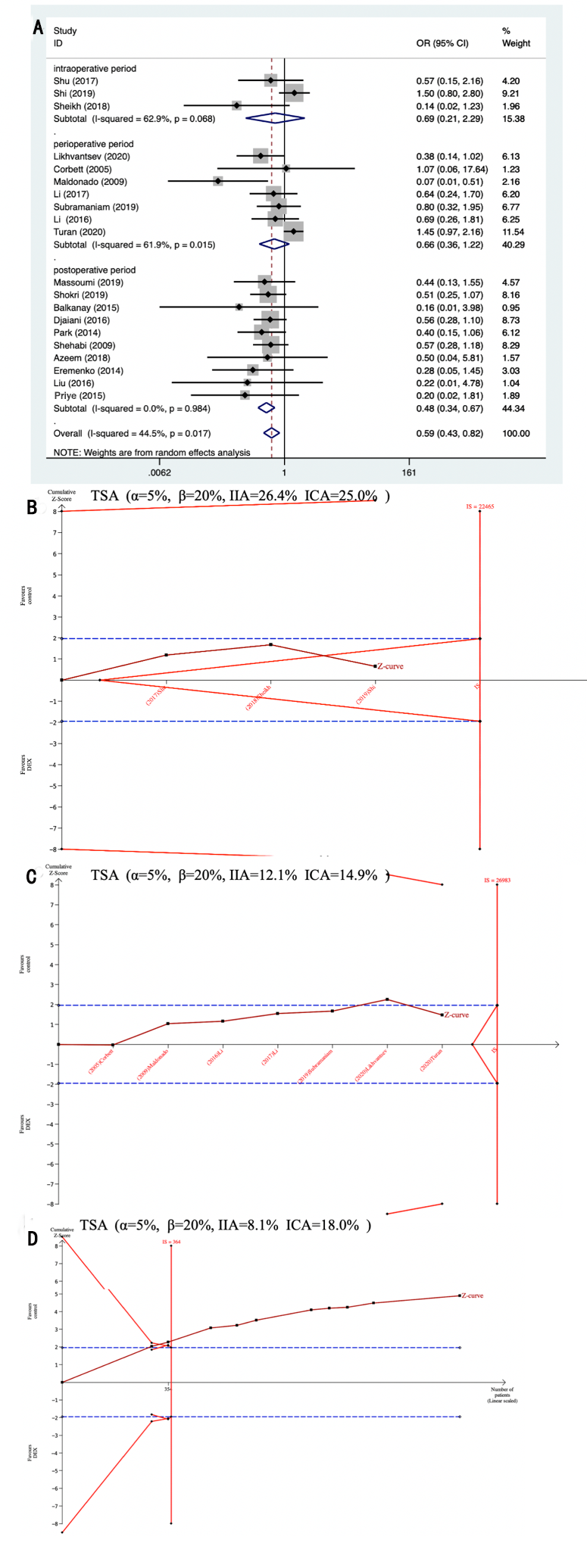
Fig S5. Postoperative delirium incidence depending on the different intervention time-point and trial sequential analyses. (A) Forest plot with subgroup analysis depending on the different intervention time-point. (B) Trial sequential analyses for ‘intraoperative period’ subgroups: error α=5%, β=20%, IIA=26.4%, ICA=25.0%. (C) Trial sequential analyses for ‘perioperative period’ subgroups: error α=5%, β=20%, IIA=12.1%, ICA=14.9%. (D) Trial sequential analyses for ‘postoperative period’ subgroups: error α=5%, β=20%, IIA=8.1%, ICA=18.0%.

NOTE: OR, odds ratio; CI, conﬁdence intervals; TSA, trial sequential analyses; IIA, incidence in intervention arm; ICA, incidence in control arm; IS, information size.


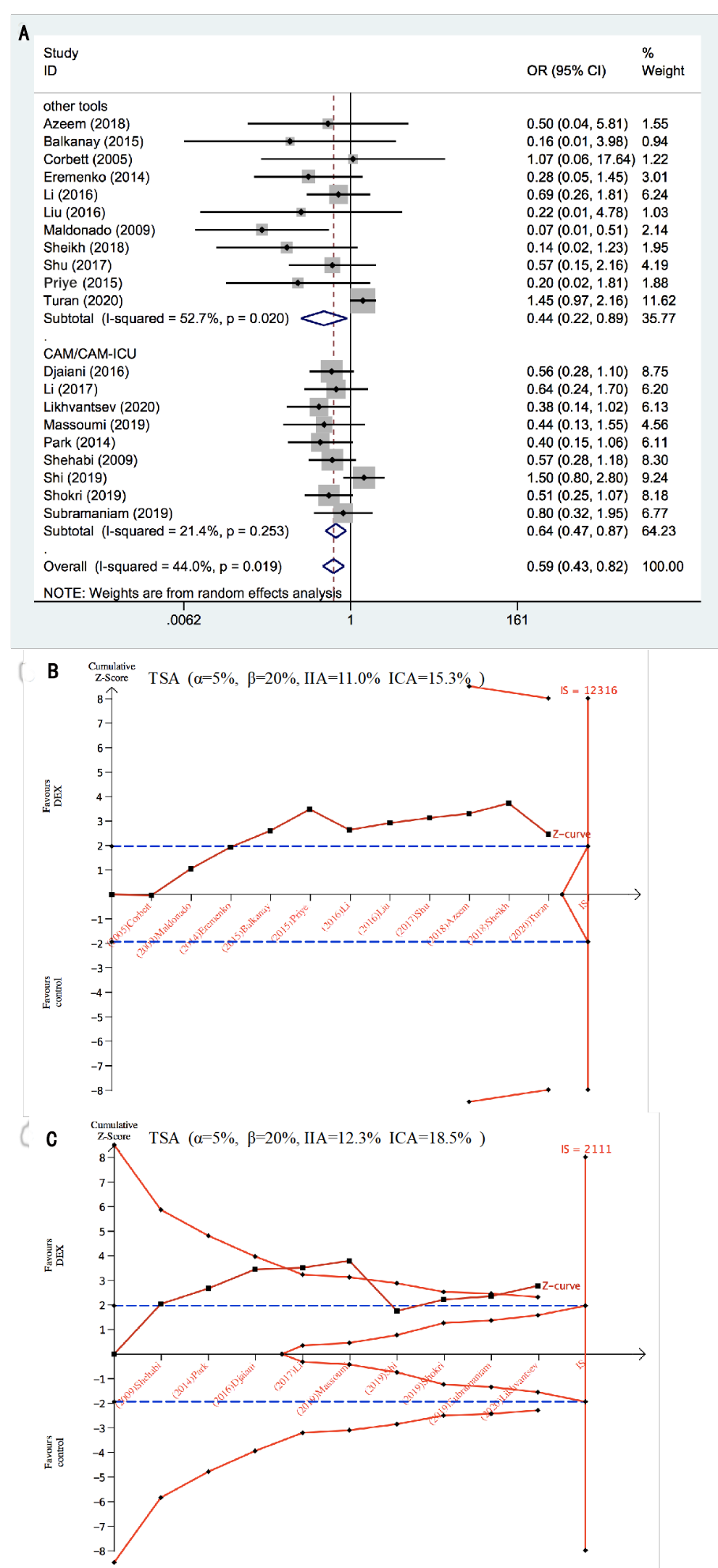
Fig S6. Postoperative delirium incidence within different diagnose tools and trial sequential analyses. (A) Forest plot with subgroup analysis depending on the different diagnose tools. (B) Trial sequential analyses for ‘other tools’ subgroups: error α=5%, β=20%, IIA=11.0%, ICA=15.3%. (C) Trial sequential analyses for ‘CAM/CAM-ICU’ subgroups: error α=5%, β=20%, IIA=12.3%, ICA=18.5%.

NOTE: OR, odds ratio; CAM, confusion assessment method; CAM-ICU, CAM in the ICU; TSA, trial sequential analyses; IIA, incidence in intervention arm; ICA, incidence in control arm; IS, information size.


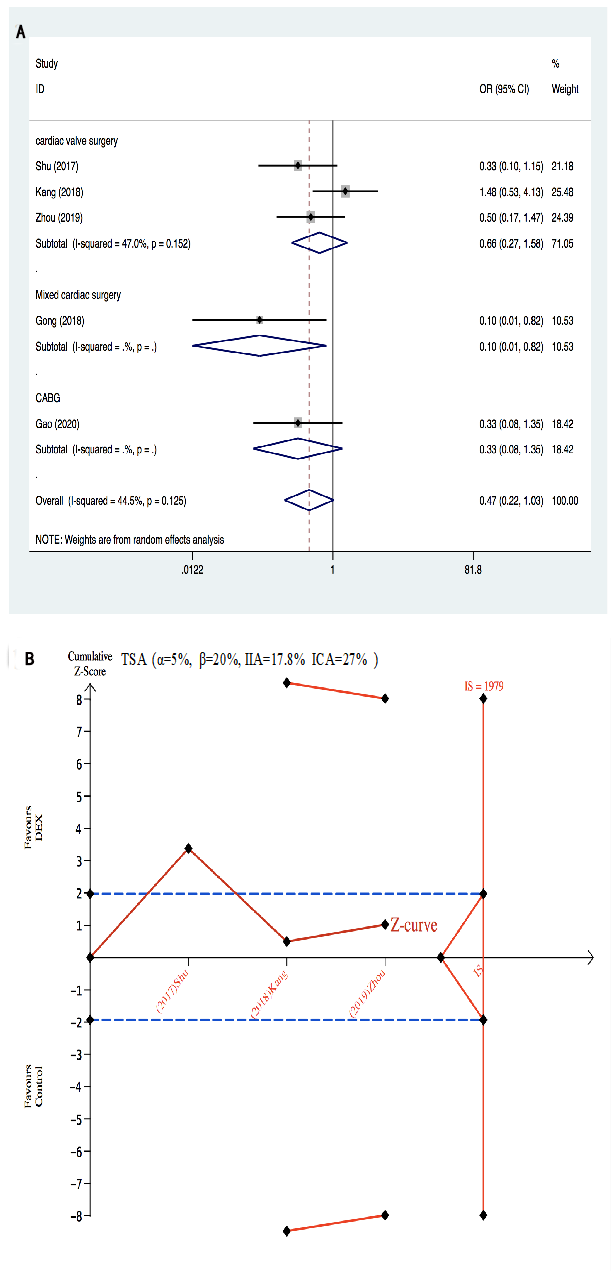
Fig S7. Postoperative cognitive dysfunction incidence depending on the type of surgery and trial sequential analyses. (A) Forest plot with subgroup analysis depending on the type of surgery. (B) Trial sequential analyses for ‘cardiac valve surgery’ subgroups: error α=5%, β=20%, IIA=17.8%, ICA=27%.

NOTE: OR, odds ratio; CABG, coronary artery bypass surgery; TSA, trial sequential analyses; IIA, incidence in intervention arm; ICA, incidence in control arm; IS, information size.


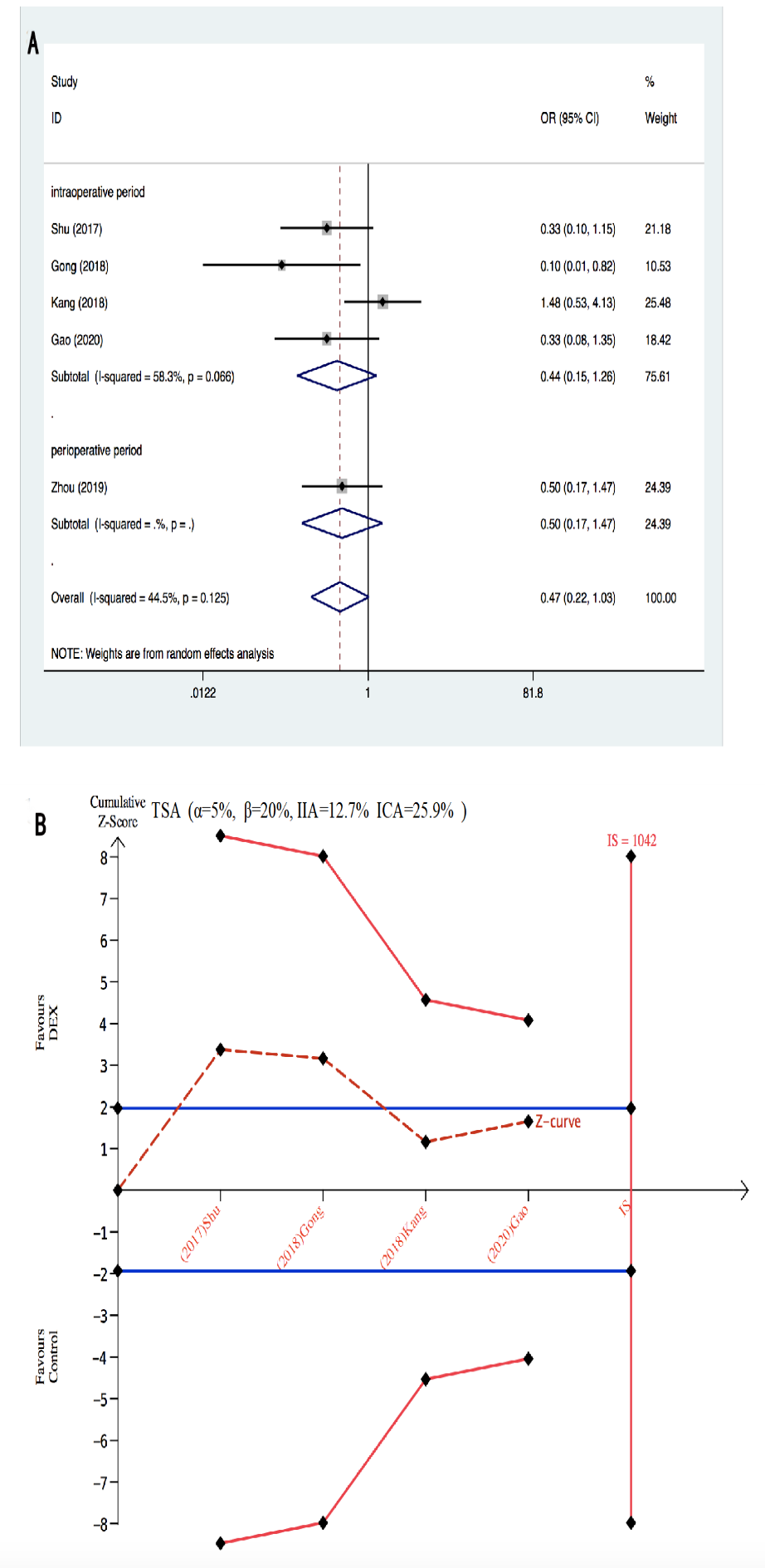
Fig S8. Postoperative cognitive dysfunction incidence depending on the different intervention time-point and trial sequential analyses. (A) Forest plot with subgroup analysis depending on the different intervention time-point. (B) Trial sequential analyses for ‘intraoperative period’ subgroups: error α=5%, β=20%, IIA=12.7%, ICA=25.9%.

NOTE: OR, odds ratio; TSA, trial sequential analyses; IIA, incidence in intervention arm; ICA, incidence in control arm; IS, information size.
