# Supplementary material for: Is Perioperative Dexmedetomidine Associated With a Reduced Risk of Perioperative Neurocognitive Disorders Following Cardiac Surgery? A Systematic Review and Meta-Analysis With Trial Sequential Analysis of Randomized Controlled Trials
Source: Front Med (Lausanne). 2021 Sep 29;8:645975. doi: 10.3389/fmed.2021.645975 (PMC8511308; doi:10.3389/fmed.2021.645975)
Supplement: Supplementary file 3 [file Data_Sheet_3.docx]

| **Supplementary 3. (A) GRADE evidence for dexmedetomidine on postoperative delirium incidence (POD).** | | | | | | |
| --- | --- | --- | --- | --- | --- | --- |
| **Patient or population:** patients with risk of POD after cardiac surgery **Intervention:** Dexmedetomidine | | | | | | |
| **Outcomes** | **Illustrative comparative risks* (95% CI)** | | **Relative effect (95% CI)** | **No of Participants (studies)** | **Quality of the evidence (GRADE)** | **Comments** |
|  | Assumed risk | Corresponding risk |  |  |  |  |
|  | **Control** | **POD** |  |  |  |  |
| **POD incidence** | **Study population** | | **OR 0.51**  (0.34 to 0.75) | 3297 (20 studies) | ⊕⊕⊕⊝ **moderate**^1^ |  |
|  | **170 per 1000** | **95 per 1000** (65 to 133) |  |  |  |  |
|  | **Medium risk population** | |  |  |  |  |
|  | **175 per 1000** | **98 per 1000** (67 to 137) |  |  |  |  |
| **type of cardiac surgery** | **Study population** | | **OR 0.5**  (0.35 to 0.73) | 3297 (20 studies) | ⊕⊕⊕⊝ **moderate**^1^ |  |
|  | **170 per 1000** | **93 per 1000** (67 to 130) |  |  |  |  |
|  | **Medium risk population** | |  |  |  |  |
|  | **175 per 1000** | **96 per 1000** (69 to 134) |  |  |  |  |
| **type of cardiac surgery - mixed cardiac surgery** | **Study population** | | **OR 0.58**  (0.38 to 0.9) | 2443 (14 studies) | ⊕⊕⊕⊝ **moderate**^1^ |  |
|  | **161 per 1000** | **100 per 1000** (68 to 147) |  |  |  |  |
|  | **Medium risk population** | |  |  |  |  |
|  | **175 per 1000** | **110 per 1000** (75 to 160) |  |  |  |  |
| **type of cardiac surgery - CABG** | **Study population** | | **OR 0.41**  (0.23 to 0.71) | 646 (5 studies) | ⊕⊕⊕⊕ **high** |  |
|  | **143 per 1000** | **64 per 1000** (37 to 106) |  |  |  |  |
|  | **Medium risk population** | |  |  |  |  |
|  | **162 per 1000** | **73 per 1000** (43 to 121) |  |  |  |  |
| **type of cardiac surgery - cardiac valve surgery** | **Study population** | | **OR 0.26**  (0.04 to 1.57) | 208 (3 studies) | ⊕⊕⊕⊝ **moderate**^2^ |  |
|  | **331 per 1000** | **114 per 1000** (19 to 437) |  |  |  |  |
|  | **Medium risk population** | |  |  |  |  |
|  | **233 per 1000** | **73 per 1000** (12 to 323) |  |  |  |  |
| **time and duration of dexmedetomidine administration** | **Study population** | | **OR 0.51**  (0.34 to 0.75) | 3297 (20 studies) | ⊕⊕⊕⊝ **moderate**^1^ |  |
|  | **170 per 1000** | **95 per 1000** (65 to 133) |  |  |  |  |
|  | **Medium risk population** | |  |  |  |  |
|  | **175 per 1000** | **98 per 1000** (67 to 137) |  |  |  |  |
| **time and duration of dexmedetomidine administration - perioperative period** | **Study population** | | **OR 0.75**  (0.41 to 1.38) | 1687 (7 studies) | ⊕⊕⊝⊝ **low**^1,3^ |  |
|  | **149 per 1000** | **116 per 1000** (67 to 195) |  |  |  |  |
|  | **Medium risk population** | |  |  |  |  |
|  | **162 per 1000** | **127 per 1000** (73 to 211) |  |  |  |  |
| **time and duration of dexmedetomidine administration - intraoperative period** | **Study population** | | **OR 0.61**  (0.14 to 2.73) | 284 (3 studies) | ⊕⊕⊕⊝ **moderate**^2^ |  |
|  | **250 per 1000** | **169 per 1000** (45 to 476) |  |  |  |  |
|  | **Medium risk population** | |  |  |  |  |
|  | **233 per 1000** | **156 per 1000** (41 to 453) |  |  |  |  |
| **time and duration of dexmedetomidine administration - postoperative period** | **Study population** | | **OR 0.4**  (0.28 to 0.56) | 1326 (10 studies) | ⊕⊕⊕⊕ **high** |  |
|  | **180 per 1000** | **81 per 1000** (58 to 109) |  |  |  |  |
|  | **Medium risk population** | |  |  |  |  |
|  | **159 per 1000** | **70 per 1000** (50 to 96) |  |  |  |  |
| **diagnostic tool** | **Study population** | | **OR 0.51**  (0.34 to 0.75) | 3297 (20 studies) | ⊕⊕⊕⊝ **moderate**^1^ |  |
|  | **170 per 1000** | **95 per 1000** (65 to 133) |  |  |  |  |
|  | **Medium risk population** | |  |  |  |  |
|  | **175 per 1000** | **98 per 1000** (67 to 137) |  |  |  |  |
| **diagnostic tool - CAM/CAM-ICU** | **Study population** | | **OR 0.48**  (0.35 to 0.65) | 1736 (9 studies) | ⊕⊕⊕⊕ **high** |  |
|  | **185 per 1000** | **98 per 1000** (74 to 129) |  |  |  |  |
|  | **Medium risk population** | |  |  |  |  |
|  | **196 per 1000** | **105 per 1000** (79 to 137) |  |  |  |  |
| **diagnostic tool – other tools** | **Study population** | | **OR 0.47**  (0.23 to 0.95) | 1561 (11 studies) | ⊕⊕⊕⊕ **high** |  |
|  | **153 per 1000** | **78 per 1000** (40 to 146) |  |  |  |  |
|  | **Medium risk population** | |  |  |  |  |
|  | **159 per 1000** | **82 per 1000** (42 to 152) |  |  |  |  |
| *The basis for the **assumed risk** (e.g. the median control group risk across studies) is provided in footnotes. The **corresponding risk** (and its 95% confidence interval) is based on the assumed risk in the comparison group and the **relative effect** of the intervention (and its 95% CI).  **CI:** Confidence interval; **OR:** Odds ratio; | | | | | | |
| GRADE Working Group grades of evidence **High quality:** Further research is very unlikely to change our confidence in the estimate of effect.  **Moderate quality:** Further research is likely to have an important impact on our confidence in the estimate of effect and may change the estimate. **Low quality:** Further research is very likely to have an important impact on our confidence in the estimate of effect and is likely to change the estimate. **Very low quality:** We are very uncertain about the estimate. | | | | | | |
| ^1^ different studies shown opposite conclusion  ^2^ the number of samples is small  ^3^ some studies may have risk of bias | | | | | | |
| **Supplementary 3. (B) GRADE evidence for dexmedetomidine on postoperative cognitive dysfunction (POCD) after cardiac surgery.** | | | | | | |
| **Patient or population:** patients with risk of POCD after cardiac surgery **Intervention:** Dexmedetomidine | | | | | | |
| **Outcomes** | **Illustrative comparative risks* (95% CI)** | | **Relative effect (95% CI)** | **No of Participants (studies)** | **Quality of the evidence (GRADE)** | **Comments** |
|  | Assumed risk | Corresponding risk |  |  |  |  |
|  | **Control** | **POCD** |  |  |  |  |
| **POCD incidence** | **Study population** | | **OR 0.37**  (0.15 to 0.96) | 373 (5 studies) | ⊕⊕⊕⊝ **moderate**^1^ |  |
|  | **270 per 1000** | **120 per 1000** (53 to 262) |  |  |  |  |
|  | **Medium risk population** | |  |  |  |  |
|  | **300 per 1000** | **137 per 1000** (60 to 291) |  |  |  |  |
| **type of cardiac surgery** | **Study population** | | **OR 0.37**  (0.15 to 0.96) | 373 (5 studies) | ⊕⊕⊕⊝ **moderate**^2^ |  |
|  | **270 per 1000** | **120 per 1000** (53 to 262) |  |  |  |  |
|  | **Medium risk population** | |  |  |  |  |
|  | **300 per 1000** | **137 per 1000** (60 to 291) |  |  |  |  |
| **type of cardiac surgery - mixed cardiac surgery** | **Study population** | | **OR 0.08**  (0.01 to 0.63) | 80 (1 study) | ⊕⊕⊝⊝ **low**^1,2^ |  |
|  | **250 per 1000** | **26 per 1000** (3 to 174) |  |  |  |  |
|  | **Medium risk population** | |  |  |  |  |
|  | **250 per 1000** | **26 per 1000** (3 to 174) |  |  |  |  |
| **type of cardiac surgery - CABG** | **Study population** | | **OR 0.26**  (0.06 to 1.08) | 60 (1 study) | ⊕⊕⊕⊝ **moderate**^1^ |  |
|  | **300 per 1000** | **100 per 1000** (25 to 316) |  |  |  |  |
|  | **Medium risk population** | |  |  |  |  |
|  | **300 per 1000** | **100 per 1000** (25 to 316) |  |  |  |  |
| **type of cardiac surgery - cardiac valve surgery** | **Study population** | | **OR 0.56**  (0.18 to 1.75) | 233 (3 studies) | ⊕⊕⊕⊕ **high** |  |
|  | **270 per 1000** | **172 per 1000** (62 to 393) |  |  |  |  |
|  | **Medium risk population** | |  |  |  |  |
|  | **316 per 1000** | **206 per 1000** (77 to 447) |  |  |  |  |
| **time and duration of dexmedetomidine administration** | **Study population** | | **OR 0.37**  (0.15 to 0.96) | 373 (5 studies) | ⊕⊕⊕⊝ **moderate**^1^ |  |
|  | **270 per 1000** | **120 per 1000** (53 to 262) |  |  |  |  |
|  | **Medium risk population** | |  |  |  |  |
|  | **300 per 1000** | **137 per 1000** (60 to 291) |  |  |  |  |
| **time and duration of dexmedetomidine administration - perioperative period** | **Study population** | | **OR 0.41**  (0.13 to 1.23) | 76 (1 study) | ⊕⊕⊝⊝ **low**^1^ |  |
|  | **316 per 1000** | **159 per 1000** (57 to 362) |  |  |  |  |
|  | **Medium risk population** | |  |  |  |  |
|  | **316 per 1000** | **159 per 1000** (57 to 362) |  |  |  |  |
| **time and duration of dexmedetomidine administration - intraoperative period** | **Study population** | | **OR 0.34**  (0.1 to 1.25) | 297 (4 studies) | ⊕⊕⊕⊕ **high** |  |
|  | **259 per 1000** | **106 per 1000** (34 to 304) |  |  |  |  |
|  | **Medium risk population** | |  |  |  |  |
|  | **275 per 1000** | **114 per 1000** (37 to 322) |  |  |  |  |
| *The basis for the **assumed risk** (e.g. the median control group risk across studies) is provided in footnotes. The **corresponding risk** (and its 95% confidence interval) is based on the assumed risk in the comparison group and the **relative effect** of the intervention (and its 95% CI).  **CI:** Confidence interval; **OR:** Odds ratio; | | | | | | |
| GRADE Working Group grades of evidence **High quality:** Further research is very unlikely to change our confidence in the estimate of effect.  **Moderate quality:** Further research is likely to have an important impact on our confidence in the estimate of effect and may change the estimate. **Low quality:** Further research is very likely to have an important impact on our confidence in the estimate of effect and is likely to change the estimate. **Very low quality:** We are very uncertain about the estimate. | | | | | | |
| ^1^ the number of samples is small  ^2^ some studies may have risk of bias | | | | | | |
